# Evaluation of the Padua Prediction Score ability to predict venous thromboembolism in Israeli non-surgical hospitalized patients using electronic medical records

**DOI:** 10.1038/s41598-022-10209-9

**Published:** 2022-04-12

**Authors:** O. Lavon, T. Tamir

**Affiliations:** 1 Clinical Pharmacology and Toxicology Unit, Carmel Medical Center, Michal St. 7, 3436212 Haifa, Israel; 2 Rappaport Faculty of Medicine, Technion-Israel Institute of Technology, Haifa, Israel

**Keywords:** Health care, Medical research, Risk factors

## Abstract

Venous thromboembolism (VTE) is considered a leading safety concern during hospitalization. The Padua Predication Score (PPS) is a risk model conceived to predict VTE among non-surgical hospitalized patients. The study aim was to evaluate the PPS ability to predict VTE in Israeli non-surgical hospitalized patients using data from electronic medical records. A single center, large-scale, historic cohort study of hospitalized non-surgical patients was conducted. Outcomes included clinically diagnosed symptomatic VTE events, bleeding events, and mortality during hospitalization and up to 90 days thereafter, and readmission up to 90 days after discharge. 5117 patient records were analyzed after screening and validation. 1120 (22%) patients were defined per PPS as high-risk, of which 277 (24.7%) were prophylactically treated. The low-risk group included 3997 (78%) patients. Prevalence of symptomatic VTE was low. Overall, 14 (0.27%) VTE events were diagnosed: 3 cases in the high-risk group (0.27%) and 11 (0.28%) in the low-risk group, with no significant difference, p = 0.768. Prophylactic treatment among the high-risk patients did not significantly improve VTE incidence: 1/277 (0.36%) treated vs. 2/843 (0.24%), p = 0.343. There was no significant difference between the study groups regarding the rates of bleeding, unexplained mortality or readmission. PPS was not found to be an efficient tool for identification of non-surgical hospitalized patients with high risk for clinically significant VTE.

## Introduction

Venous thromboembolism (VTE) is a disorder in which thrombi are formed in deep veins and either result in in-situ occlusion of the veins (deep vein thrombosis; DVT) or detach and spread to other contiguous veins and/or embolize, including to pulmonary arteries (pulmonary embolism; PE)^1^.

Each year globally a population of over 10 million develop VTE with an annual mortality toll of 100 to 300 thousand in the USA alone^2^. Hospitalization is regarded as a major risk factor for VTE morbidity and mortality^3^. The inpatient population with the highest risk for VTE is post-operative, mainly orthopedic patients. The risk for VTE in non-surgical hospitalized patients is vaguer and depends on risk assessment. While in the past, prophylactic treatment was a universal practice in mainly all inpatients including non-surgical, current guidelines recommend performing risk stratification upon hospital admission using various scoring methods^3^. Several assessment tools for non-surgical patients are available, including Padua, Kucher, Caprini, Geneva, IMPROVE and 4-elements^4^. Each of these tools has its advantages and limitations; none is significantly preferred over the others. Padua Prediction Score (PPS) is one of the accepted scoring methods for VTE risk assessment of non-surgical patients during hospitalization and immediately following it^5^. PPS is based on 11 criteria, each with its own score. The accumulated score determines the level of risk. A score of 4 and above indicates higher risk and a recommendation for prophylactic measures, mainly anticoagulants. Table 1 presents the PPS criteria and scale. The clinical adequacy of PPS was evaluated by Barber et al. in a cohort of 1180 Italian non-surgical hospitalized patients (the Padua study)^5^. During this study, VTE incidence was 11% in the non-treated, higher risk population (n = 283) when using PPS, compared to only 0.3% in lower risk patients (n = 711). VTE incidence was decreased to 3% in prophylactically treated high risk patients (n = 186). Nevertheless, several studies have reported that only 30–40% of these high-risk patients are prophylactically treated^6–9^.

**Table 1:** Padua Prediction Score.

Baseline features	Score
Active cancer (local or distant metastases; chemotherapy and/or radiotherapy in the last 6 months)	3
Previous VTE (with exclusion of superficial vein thrombosis)	3
Reduced mobility (bedrest with bathroom privileges for at least 3 days)	3
Already known thrombophilia	3
Recent trauma and/or surgery in the last month	2
Age ≥ 70	1
Heart and/or respiratory failure	1
Acute myocardial infarction or ischemic stroke	1
Acute infection and/or rheumatologic disorder	1
Obesity (BMI ≥ 30)	1
Ongoing hormonal treatment	1

In Israel, since 2014, PPS is used as a national compulsory assessment tool for non-surgical hospitalized patients. Adherence to this assessment was determined as a national health quality measure and it is systematically reviewed by the Israel Ministry of Health. Following this national program, adherence of VTE risk assessment upon admission to hospital of non-surgical patients increased to over 80%^10^. The clinical effect of increased use of PPS on VTE incidence in the above patients is unclear.

The objective of this study was to evaluate the ability of PPS to predict VTE in Israeli non-surgical hospitalized patients by following the Padua study outline and using electronic medical records.

## Results

**Figure 1:**
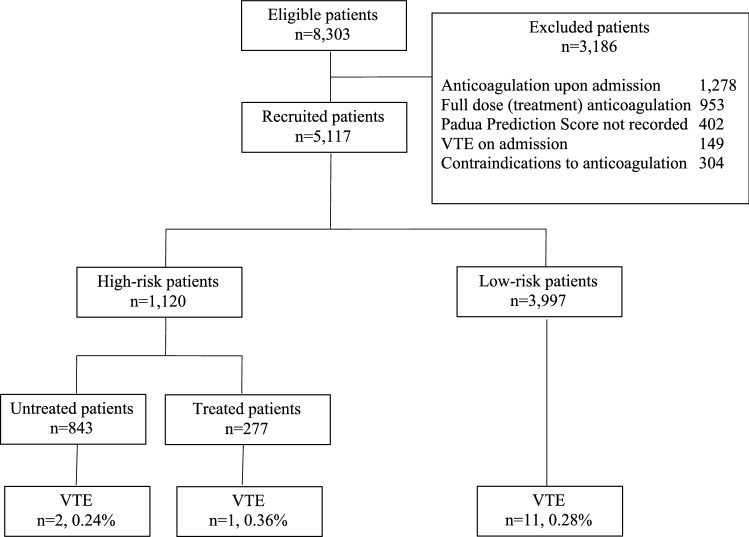
Study outline.

Of 8303 hospitalized non-surgical patients during the study period, 3186 were excluded based on the exclusion criteria. Excluded patients included 1287 on prior regular anticoagulation, 1002 that started full anticoagulation on admission due to coronary or VTE events, 304 with contraindication for anticoagulation, 268 were younger than 40 years of age and 234 without PPS estimation on admission (Fig. 1).

After exclusion, 5117 patient records were analyzed. Mean age of the patients was 74.1 ± 12.5 years; 54.2% were male. The cohort was divided, based on the Padua study design, to the different risk groups (Fig. 1). Table 2 presents the characteristics of patients in the different groups. 3,997 patients had PSS < 4 that indicated a low risk for VTE and 1120 patients with PSS ≥ 4 indicating a high risk. Prophylactic anticoagulant treatment (hospital protocol, Enoxaparin 20–40 mg QD) was given to 277 (24.7%) high risk patients.

**Table 2:** Patients characteristics.

	Low-risk	High-risk			All n = 5117
	Total n = 3,997	Untreated n = 843	Treated n = 277	Total n = 1120	
Age in years mean (SE)	72.6 (13.8)	80.1 (11.5)	78 (11.2)	79.6 (11.4)	74.1 (12.5)
Gender, male %	53.2	56.5	62.2	57.9	54.2
Hospital stay in days mean (SE)	5.9 (7.5)	7.1 (9.7)	13.8 (18.1)	8.8 (12.6)	6.53 (10.1)
PPS median, mean (SE)	1, 1.1 (0.82)	5, 5.4 (2.07)	5, 5.75 (2.05)	5, 5.4 (2.06)	1, 2.07 (2.2)
Active cancer No. (%)	83 (2.07)	25 (31.4)	66 (23.8)	331 (29.5)	414 (8.1)
Previous VTE No. (%)	13 (0.3)	158 (18.7)	63 (22.7)	221 (19.7)	234 (4.5)
Immobility No. (%)	32 (0.8)	558 (66.2)	217 (78.3)	775 (69.2)	807 (15.8)
Thrombophilia No. (%)	8 (0.2)	144 (17)	49 (17.7)	193 (17.2)	201 (3.9)
Surgery or trauma in the last month No. (%)	42 (1)	80 (9.5)	36 (13)	116 (10.3)	158 (3)
Age > 70 years No. (%)	2389 (60)	704 (83.5)	227, 82	931 (83.1)	3320 (64.9)
Cardiac or respiratory failure No. (%)	336 (8.4)	260 (30.8)	98 (35.3)	358 (32)	694 (13.6)
Myocardial infarction or stroke No. (%)	79 (2)	88 (10.4)	22 (8)	110 (10)	189 (3.7)
Acute infection or rheumatic disease No. (%)	327 (8.2)	245 (29.1)	92 (33.2)	337 (30)	664 (12.9)
Obesity No. (%)	788 (20)	206 (24.4)	67 (24.2)	273 (24.4)	1061 (20.7)
Hormonal treatment No. (%)	36 (0.9)	48 (5.7)	10 (3.6)	58 (5.2)	94 (1.8)

Overall, only 14 (0.27%) cases of diagnosed symptomatic VTE were recorded during hospitalization or up to 90 after discharge; 3 (0.27%) out of 1120 high risk patients (1 DVT, 2 PE) and 11 (0.28%) out of 3997 low risk patients (3 DVT, 8 PE). No statistically significant difference was found between risk groups regarding VTE incidence, Adjusted Risk Ratio (aRR) = 1.292, CI95% 0.237–7.052, p = 0.768. The calculated predictability value of PPS per the statistical model was low, R^2^ = 0.046.

No statistically significant difference was found between prophylactically treated and not treated high risk patients regarding VTE incidence: 1/277 (0.36%) treated vs. 2/843 (0.24%) not treated, aRR = 0.432, CI95% 0.076–2.45, p = 0.343.

Major bleedings were relatively rare; 3 (0.23%) events were recorded in PPS high risk patients, with no significant difference between treated (n = 1, 0.36%) and not treated (n = 2, 0.24%).

Mortality during hospitalization and up to 90 days later was 15% (770 cases). Almost two thirds of them (n = 438, 62%) have died during hospitalization. While overall, mortality among high risk patients was higher compared to low risk (n = 363, 32.4% *vs.* n = 407, 10.4%), there was no significant difference between the groups regarding sudden or otherwise unexplained death (n = 2, 0.17% *vs.* n = 4, 0.1%, p = 0.862). Independent risk factors for mortality were older age, obesity, heart or respiratory failure and longer hospitalization. No significant difference was found between treated and non-treated high-risk patients (p = 0.486).

Readmission rates up to 90 days after discharge were similar between risk groups (12.5% *vs.* 11.2%, p = 0.513). No significant difference in readmission was found between prophylactically treated and non-treat patients (p = 0.403).

## Discussion

We evaluated the ability of the PPS to predict VTE in non-surgical hospitalized patients. The results did not demonstrate significant benefit of the PPS or the prophylactic anticoagulation based on it. The occurrence of recorded clinically significant VTE events in non-surgical patients during hospitalization and up to 90 days thereafter was relatively low, about 1 event in 400 admissions, compared to previously reported estimations^5,11^.

The study results demonstrated a difference of 0.01% between the risk groups (0.27% vs. 0.28%) and the statistical analysis indicated that this difference in the studied sample size of about 15,000 patients is not statistically significant. The calculated sample size to show statistical significance of a difference of 0.1% (tenfold higher than the observed difference) is about 80,000 patients and for a difference of 0.01% (as was observed in the study) it is over 8 million patients. This means that a profoundly high number of patients would have to be treated to prevent a single VTE case, which exclude any clinical significance. The potential harm and high cost dramatically outweigh any benefit in this case.

The use of PPS was also not related to improved or worsen safety regarding bleeding events. The routine performance of PPS and the implementation of pharmacologic prophylaxis following PPS estimations involve substantial resources (medical personnel time, medication cost) and may distract physicians. Lack of beneficial effects calls to consider discontinuation of this practice to save resources.

While the presented study followed the Padua study outline, there is a marked difference between the VTE incidences observed in the studies, over tenfold. The diagnosis of VTE events in the Padua study was mainly based on positive imaging and laboratory (d-dimer) results and not on clinical symptoms^5^. A high percentage of the Padua study participants was referred to testing. Data regarding the clinical significance of the VTE diagnosis in the Padua study are limited. Contrary, the presented study identified recorded symptomatic clinically meaningful VTE events that demanded intervention or readmission. The different methods may have led to the different outcome rates. In addition, the duration of hospitalization was relatively longer in the Padua study, which may have contributed to the elevated VTE prevalence.

Others also found low efficiency of the PPS. Saliba et al. measured single thrombin generation in acutely hospitalized patients and found no correlation with the PPS^12^. Vardi et al. showed that PPS estimation lacks granularity in detecting non-surgical septic patients at risk of acquiring VTE^13^. Depietri et al. assessed the application of risk assessment models, including the PPS, on VTE and major hemorrhage on internal medicine hospitalized patients and found it statistically ineffective^14^.

The PPS was compared to other risk assessment tools. The Caprini risk assessment model (RAM) performed better than the PPS in 2 studies. Zhou et al. assessed the validity of the Caprini RAM in Chinese hospitalized patients with VTE and compared it to the Kucher tool and the PPS^15^. Caprini model was found to classify much more VTE patients into high or highest risk level than the other models with statistically significant differences (Caprini model *vs* Kucher model, p < 0.0001; Caprini model *vs* the PPS, p < 0.0001). In another study by Zhou et al. the Caprini RAM was compared again to the PPS^16^. The Cparini RAM defined 82.3% of VTE cases as high risk, while the PPS has defined only 30.1% of these same cases. Nendaz et al. investigated the Geneva risk score among Swiss hospitalized medical patients^17^. They found that for VTE prediction, the Geneva Risk Score compared favourably with the PPS, particularly for its accuracy to identify low-risk patients who do not require thromboprophylaxis. The main practical limitation of the Caprini and Geneva models is their relative complexity, as they include 39 and 19 criteria, respectively, compared to the 11 criteria of PPS.

PPS is a mathematical scoring method based on the models of Kucher et al. and Lecumberri et al. that uses some of the criteria in the original models^18,19^. Its ability to capture the full clinical complexity of different patients and become a globally generalized valid assessment tool is limited. Risk assessment should be comprehensive and appropriately tailored to the patients and the relevant population. In addition, risk should be continuously evaluated and not assessed only at a single time point, such as admission.

The issue of any VTE diagnosis *vs.* symptomatic VTE is paramount. The practical contribution of asymptomatic VTE diagnosis to patients’ health and prognosis can be debated. Hospital related VTE was considered a safety concern following 2 studies published in the late 1980s^20,21^. Both were based on autopsies of patients that died during hospitalization or shortly after. These studies only demonstrated the presence of VTE in the body; there was no proof of causality or any linkage between VTE post-mortem presence and either morbidity or mortality. The Worchester study from 1991 showed higher VTE rates in hospitalized patients compared to community; this study also reported mainly asymptomatic cases^22^. About 10 years later (2002–2003), 3 large-scale clinical trials sponsored by pharmaceutical companies were published almost simultanesly^23–25^. They all demonstrated significant improvement in VTE incidence following prophylactic anticoagulants. A critical review of the studies by Vardi and Haran emphasized that most of the VTE cases in the clinical trials were asymptomatic^26^. Symptomatic VTE rates were low and there was no benefit for the prophylaxis when observing only symptomatic cases.

During the years, there were several other publications that supported the importance of VTE risk during hospitalization and immediately after but based their conclusion on previous papers that mainly cited the old autopsy studies. Pradoni—coauthor of the Padua study—cited Leizorovicz and Mismetti to support in his paper the claim regarding VTE elevated risk in non-surgical hospitalized patients^27,28^. Leizorovicz and Mismetti themselves have based this claim on the THRIFT study paper^29^, which provides as references the 1980s autopsy studies, again^20,21^. The claim that the risk for clinically meaningful VTE in non-surgical hospitalized patients is significantly elevated, is poorly substantiated on clear clinical data.

Diagnosis of asymptomatic VTE is increasing with the advances in diagnostic technologies. Computed tomography pulmonary angiography is able to pick up pulmonary emboli as small as 2–3 mm in diameter, but their clinical relevance is questioned^30,31^. Unnecessary anticoagulation increases the risk for major bleedings^32^.

The pathophysiology of VTE is related to an imbalance of 3 components of the Virchov’s triangle: hypercoagulability, stasis, and endothelial impairment^33^. This imbalance does not necessarily worsen upon admission to hospitalization for most non-surgical patients; it can even improve. The risk for VTE is inherent and relatively fixed due to the patient’s prolonged personal medical and functional status before admission. Many of these patients are functionally impaired and immobile long before their hospitalization, while immobility of hospitalized patients is usually properly addressed by repeated position changes and other means and procedures, as instructed by current guidelines and hospital accreditation standards^34^. Blood viscosity is improved upon admission of deteriorated or dehydrated patients using fluid therapy. Infection and inflammation are appropriately and rapidly managed in most admitted patients. These interventions during hospitalization, which are nowadays a common practice, reduce the risk for VTE and the need for pharmacological prophylaxis. Shortening of the hospitalization is another key element of mitigating the risk.

Recent studies have indicated a low incidence of symptomatic VTE in hospitalized non-surgical patients. Fritz et al. found that only 0.3% of general medicine hospitalized patients developed VTE^35^. Koren et al. evaluated over 500 Israeli medical hospitalized patients and did not find any hospital acquired symptomatic VTE^36^. Vardi et al. studied septic patients in Israeli internal medicine departments and found a low rate of symptomatic VTE^13^. Kolomansky et al. evaluated prospectively the occurrence of VTE in Israeli hospitalized patients. Overall, VTE was diagnosed in 0.25% of these patients^37^. These reports support the presented study results.

Another support to the low incidence of clinically significant VTE in hospitalized patients comes from a recently published OECD report on VTE rates^38^. The recorded multinational multicenter VTE hospital-related diagnosis is 0.3% (189.3 DVT cases and 175.3 PE cases of 100,000 discharges), similar to the present study results. It represents the true extent of the clinically meaningful VTE cases.

The study has limitations. It is retrospective and based of computerized retrieval of digital data. It is depended on the heterogenic quality of medical information recorded by numerous staff members. It is a single center study and the generalization of its conclusion should be done carefully.

Nevertheless, the study holds several strengths. It has a large patients’ sample, over 5,000. More than twice the sample of the Padua study, which was also a single center study. This study outline replicated the Padua study group design and patient selection to enable a fair chance for comparison. The study period is relatively long, over 2 years, and the outcomes were measured up to 90 days after discharge. The data were pooled from 2 medical databases providing comprehensive information from the hospital and community clinics; this gives high credence to the results. An independent focused validation was performed to ensure the appropriateness of the VTE diagnosis.

In conclusion, PPS was not found to be an efficient tool for identification of non-surgical hospitalized patients with high risk for clinically significant VTE events. Prophylactic anticoagulation based on PPS did not provide significant clinical benefit. The study results indicate the need to consider stopping the use of PPS as a mandatory or preferred assessment tool for VTE in Israeli non-surgical hospitalized patients. Until confirmed, better and efficient assessment tools can be implemented, it is recommended to clinically assess VTE risk and the need for prophylaxis in hospitalized non-surgical patients on a case by case basis.

## Methods

This was a single center, large-scale, historic cohort study of hospitalized non-surgical patients in medicine and geriatric departments during a period of 26 months (April 1, 2014 to May 31, 2016) when PPS assessment was mandatory upon admission. The study outline correlated with the Padua study design. All electronic records of adult patients over the age of 40 years were retrieved and screened for eligibility. Exclusion criteria included patients with a VTE diagnosis upon admission or up to 48 h after admission (to exclude pre-admission VTE), patients with chronic anticoagulant treatment, and patients with contraindication for anticoagulants including any active bleeding, thrombocytopenia upon admission, INR over 1.5 upon admission, and advanced liver disease. Medical record retrieval and exclusion criteria screening were based on a computerized query in 2 combined databases of hospital and community records using key words and ICD-9 coding. Both databases belong to the same health maintenance organization and are fully accessible to internal authorized data-mining experts. The databases encompass medical information for the all duration of the hospitalization and 90 days following discharge. The eligible records were digitally surveyed for person-level demographic and clinical variables. Linkage of data from the 2 databases was based on each patient national identification number and allowed the creation of a unified data set for each study participant. Outcomes included diagnosis of VTE, DVT, or PE during hospitalization and up to 90 days following it, identified using key words and ICD-9 coding as pulmonary embolism (ICD-9 code 415.1x), lower extremity DVT (451.1x, 451.2, 451.81, 453.4x, 453.5x), upper extremity DVT (451.83, 451.84, 451.89, 453.72, 453.73, 453.74, 453.75, 453.76, 453.77, 453.82, 453.83, 453.84, 453.85, 453.86, 453.87), and other venous thrombosis (451, 451.9, 452, 453, 453.0, 453.1, 453.2, 453.3, 453.79, 453.8, 453.89, 453.9); prophylactic treatment with anticoagulants, identified by designated search for specific medications and doses per protocol in the Computerized Provider Order Entry (CPOE) module of the electronic medical record; any bleeding and major bleeding (as defined in the Padua study protocol^5^) during hospitalization and up to 90 days following it; readmission up to 90 days and death during hospitalization and up to 90 days following it.

VTE diagnosis was further validated using an independent assessment of each record by an internal medicine specialist. This validation process excluded non-relevant cases and guaranteed the accuracy of the VTE patient group. Other recorded variables included age, gender, comorbidities, cause of admission, duration of hospitalization, VTE risk factors (per PSS) and PSS upon admission.

Screened and validated data were transferred to an electronic data sheet (Microsoft Excel, Microsoft Office 365). Recorded data were subjected to descriptive and comparative statistical analysis using a designated software (SPSS Statistics version 27, IBM^®^, Armonk, New York, USA). Multivariate logistic regression was used to identify the predictors of VTE events, mortality or re-admission within 90 days post hospitalization and detect significant differences in the outcomes between high *vs.* low risk patients, and prophylactically treated *vs.* non-treated patients. Adjusted Risk Ratios (aRR) with 95%CI are presented; p < 0.05 was considered statistically significant. Variables included in the regression model were age, gender, previous VTE, reduced mobility, thrombophilia, recent trauma or surgery, heart or respiratory failure, acute myocardial infarction, ischemic stroke, acute infection or rheumatologic disorder, obesity and hormonal treatment. Explained predictability of each predictor was calculated and presented as R^2^. Model fitness was confirmed using Hosmer and Lemeshow test.

RECORD (REporting of studies Conducted using Observational Routinely-collected Data) guidelines were implemented during the preparation of the study report^39^.

### Ethics approval

The study was performed in accordance with relevant guidelines and regulations. The study was approved by Carmel Medical Center Institutional Review Board, approval number 16-0185. The Institutional Review Board has given waiver for the need for informed consent, as the study is retrospective, and it analyzed aggregated, unidentified data.
